# Joint Modelling Application to Identify Key Determinants of Malnutrition and Anemia in Five West African Countries

**DOI:** 10.3390/nu18203423

**Published:** 2026-10-19

**Authors:** Reshav Beni, Shaun Ramroop, Faustin Habyarimana

**Affiliations:** School of Mathematics, Statistics and Computer Science, University of KwaZulu-Natal, Pietermaritzburg 3209, South Africa

**Keywords:** malnutrition, anemia, Western Africa, joint model, public health

## Abstract

Objectives: In Sub-Saharan Africa, anemia significantly contributes to infant mortality and morbidity, particularly among children affected by malnutrition. Given the intertwined nature of these conditions, separate modeling approaches are insufficient for capturing their combined effects. Joint modeling is essential to concurrently analyze anemia and malnutrition, enabling more accurate parameter estimation and a comprehensive understanding of shared risk factors. This study investigates determinants of both conditions across select West African countries, including Gabon, Gambia, Liberia, Mauritania, and Nigeria, with the aim of guiding policy measures to reduce prevalence. A novel aspect of this research is the joint modeling framework developed to analyze anemia and malnutrition together, an approach that remains limited in existing literature. By incorporating data from multiple countries, this study provides a unique and regionally contextualized perspective, addressing critical gaps in policy-relevant research. Design: A multivariate joint model was used to identify significant predictors of anemia and malnutrition in children under five years of age. Data Source: This secondary data analysis utilized demographic and health survey (DHS) data collected in selected West African countries. The DHS applied a complex, multistage sampling design incorporating stratification and cluster sampling, ensuring that the resulting weighted samples accurately represented various population subgroups. Results: The model results identified several statistically significant determinants (*p* < 0.05) of malnutrition and anemia. These factors included the mother’s age, the highest level of maternal education, source of drinking water, type of toilet facility, household electricity access, type of cooking fuel used, household wealth index, whether the child was given fortified food, the child’s age and gender and whether the child had a fever in the past two weeks. Conclusions: This study highlights the essential role of primary healthcare interventions, community outreach, and policy initiatives in mitigating malnutrition and anemia in West African nations. Key measures include routine anemia screening for high-risk mothers, implementing standardized treatment protocols, and addressing environmental risk factors. Partnerships with local governments and nonprofit organizations are vital for providing vulnerable groups with access to fortified foods, nutritional support, and essential healthcare services. Additionally, mobile health tools and educational webinars can address literacy barriers and enhance antenatal care. A comprehensive approach incorporating education, healthcare, and technology is crucial for improving health outcomes and reducing the prevalence of these conditions.

## 1. Introduction

In Sub-Saharan Africa, anemia is found to be a significant complication contributing to high levels of infant mortality and morbidity in children diagnosed with malnutrition [1,2].

The World Health Organization (WHO) defines malnutrition as “deficiencies or excesses in nutrient intake, imbalance of essential nutrients or impaired nutrient utilization” [3]. Malnutrition encompasses both undernutrition and overnutrition (obesity) and can lead to various dietary-associated non-communicable disorders. According to the 2025 UNICEF/WHO/World Bank Joint Child Malnutrition Estimates, approximately 150.2 m children under five years of age were affected by stunting, 42.8 m by wasting, and 35.5 m by overweight in 2024. Despite the progress that has been made over recent decades, the world remains off track to achieve the Sustainable Development Goal targets for ending all forms of malnutrition by 2030. Furthermore, nearly half of deaths among children under five are linked to undernutrition, with most occurring in low- and middle-income countries [4]. This trend is particularly pronounced in West Africa, where malnutrition prevalence remains high due to a complex interplay of individual, social, economic, political, and environmental factors [5].

Anemia, as defined by WHO, is a condition characterized by a reduction in the total number of red blood cells (RBCs) or hemoglobin levels in the blood, impairing the blood’s capacity to transport oxygen to tissues and organs [6]. Anemia remains a substantial global public-health problem among young children. Children under five years of age are especially vulnerable to anemia because their rapid physical and cognitive development increases the demand for iron and other essential micronutrients. Inadequate dietary intake, poor nutrient absorption, and recurrent infections may further increase their risk of developing anemia [7].

The most recent WHO global estimate indicates that 39.8% of children aged 6–59 months were affected by anemia in 2019, equivalent to approximately two in every five children in this age group [8]. In infancy and early childhood, anemia is associated with impaired cognitive development, growth retardation, weakened immune function, and increased mortality risk [1]. Recognizing its severe health implications, WHO designated anemia as a global public health concern in 2004 [9].

Previous studies have examined the determinants of childhood anemia and malnutrition, but these outcomes have frequently been analyzed separately, despite their potential to coexist and share common socioeconomic, demographic, environmental, and health-related determinants. In addition, much of the existing evidence has been based on single-country analyses, limiting the ability to identify determinants that are common across countries while accounting for country-specific differences. Consequently, relatively little is known about the factors associated with the joint occurrence of anemia and malnutrition across multiple West African countries.

Anemia and malnutrition are biologically interconnected through several pathways. Inadequate dietary intake may lead to deficiencies in iron, folate, vitamin B12, vitamin A, and other micronutrients required for haemoglobin synthesis and erythropoiesis [10]. Malnutrition may also impair intestinal function and nutrient absorption, thereby reducing the bioavailability of iron and other haemopoietic nutrients. Furthermore, undernourished children may experience impaired immune function and increased susceptibility to infections. Infection-induced inflammation stimulates the production of hepcidin, which reduces intestinal iron absorption and restricts the release of stored iron into the circulation, thereby limiting the iron available for red blood cell production. In malaria-endemic settings, malaria may further contribute to anemia through red blood cell destruction and impaired erythropoiesis, while helminth infections may cause chronic blood loss and interfere with nutrient absorption [11]. These mechanisms may create a reinforcing cycle in which malnutrition increases susceptibility to anemia, while anemia may contribute to reduced appetite, diminished physical capacity, and impaired growth and development. Consequently, the two conditions may share nutritional, infectious, biological, and socioeconomic determinants, supporting their simultaneous assessment within a joint modelling framework.

The present study addresses these gaps by developing a joint modelling framework to analyze anemia and malnutrition concurrently among children in Gabon, The Gambia, Liberia, Mauritania, and Nigeria. By accounting for the dependence between the two outcomes and pooling evidence across multiple countries, this study provides a more integrated assessment of their shared and outcome-specific determinants. This multi-country approach also makes it possible to examine whether observed associations are consistent across different national contexts. The findings are intended to support the development of more coordinated and context-sensitive policies and interventions aimed at reducing the burden of anemia and malnutrition in West Africa.

## 2. Data

The analysis was based on secondary data from Demographic and Health Surveys undertaken across five selected African countries. A complex, two-stage probability sampling procedure was used in each survey, combining stratification and cluster sampling. As participants had unequal probabilities of being selected, survey weights were incorporated into the analysis to account for the sampling design and support the calculation of nationally representative estimates.

Guided by the DHS data-management manual, the study variables were extracted from the Kids Recode files. Within these files, each record corresponds to a child younger than five years born to a woman who was interviewed during the survey. The separate country-level files were harmonised and vertically appended to form a pooled dataset consisting of observations from Gabon (2019–2021), The Gambia (2019–2020), Liberia (2019–2020), Mauritania (2019–2021), and Nigeria (2021).

Child nutritional status was evaluated using stunting and anemia as the primary outcome measures. Stunting represents the cumulative effects of chronic undernutrition and was determined for children younger than 59 months based on their height-for-age z-scores. For the analysis, HAZ values below −2 standard deviations were coded as stunted, while values equal to or above −2 standard deviations were coded as not stunted. Anemia status was similarly treated as a binary outcome indicating whether a child was anemic or non-anemic [12].

Stunting was selected as the primary indicator of malnutrition because it captures long-term nutritional deprivation, which aligns with the study’s objective of examining structural and persistent determinants of child health. Unlike wasting (WHZ < −2.0) and underweight (WAZ < −2.0)—which reflect acute and mixed forms of undernutrition, respectively—stunting is less sensitive to short-term fluctuations and more indicative of chronic exposure to adverse conditions such as poverty, inadequate dietary diversity, and poor sanitation. This makes it a more stable and policy-relevant measure for assessing the cumulative impact of socioeconomic and environmental factors on child development. For the analysis, stunting status was represented as a binary variable, with children whose height-for-age z-scores were below −2.0 standard deviations coded as 1 and those with scores of −2.0 standard deviations or higher coded as 0. The −2.0 SD threshold is an internationally accepted criterion for identifying stunting among children [1]. Applying this threshold provides a consistent and standardised approach to measuring chronic undernutrition in clinical and population-based research [13]. In accordance with the World Health Organization classification, a child was classified as having moderate-to-severe anemia if their altitude-adjusted haemoglobin concentration was below 10.0 g/dL, encompassing moderate anemia (7.0–9.9 g/dL) and severe anemia (<7.0 g/dL). Children with an altitude-adjusted haemoglobin concentration of 10.0 g/dL or higher were classified as not having moderate-to-severe anemia and constituted the comparison group. The outcome was coded as 1 for moderate-to-severe anemia and 0 otherwise [14].

Potential explanatory variables were selected based on demographic, socioeconomic, environmental, healthcare, dietary, and child health factors previously associated with childhood malnutrition and anemia. The demographic characteristics considered were the respondent’s region, maternal age, place of residence, maternal educational attainment, the sex and age of the household head, and the child’s age and sex. Household socioeconomic and environmental characteristics included household wealth, access to electricity, drinking-water source, type of sanitation facility, cooking fuel, and the availability of a mosquito net.

Healthcare and malaria-prevention factors comprised the respondent’s use of a mosquito net on the night preceding the survey and attendance at a health facility during the previous 12 months. Child-feeding indicators captured whether the child had consumed milk, infant formula, fortified foods, potatoes, cassava or other tubers, eggs, and meat. The child-level health measures included anemia severity and whether the child had experienced fever or cough during the two weeks preceding the survey.

## 3. Model Outline

The multivariate analysis was conducted using the PROC GLIMMIX procedure in SAS 9.4, which facilitated the joint modeling of two outcome variables sharing similar distributions and link functions. In this study, both outcomes were assumed to have identical distributions and link functions, optimizing interpretability and ensuring a consistent model structure. Various covariance structures were evaluated to identify the most appropriate fit, with model suitability assessed using the Akaike Information Criterion (AIC). The Unstructured (UN) covariance structure was determined to be the most suitable based on the lowest AIC value, providing a flexible approach to account for dependencies in the data. Interactions between the outcome indicator and each explanatory variable were tested to determine whether the associations differed between anemia and malnutrition. None reached statistical significance at the 5% level; therefore, common effects across the two outcomes were retained in the final model, suggesting independent contributions of the main effects. This analytical approach allowed for a robust investigation of determinants while maintaining parsimony and interpretability in the model outcomes [15,16,17].

## 4. Methodology

Consider $Y_{ij}$ as a binary random variable, denoted by values 1 or 0, representing the $i^{th}$ outcome (where i = 1, 2) of the $j^{th}$ subject (where j = 1, 2, …, n). Here, i = 1 indicates nutritional status, and i = 2 indicates anemia status.

The two binary outcomes together form a bivariate binary response vector $Y=\left(Y^{1j}|Y^{2j}\right)$. If nutritional status and anemia status are independent, the generalized linear mixed model is expressed as follows [2]:

(1) $$g_{1}\left(\mu_{1j}\right)\;=\;B_{1}X_{1j}+Z_{1j}\gamma_{1j}+\;\varepsilon_{1j}$$

(2) $$g_{2}\left(\mu_{2j}\right)=B_{2}X_{2j}+Z_{2j}\gamma_{2j}+\varepsilon_{2j}$$

where

$\varepsilon_{1i}\sim N(0,\sigma_{1}^{2})$,

$\varepsilon_{2i}\sim N(0,\sigma_{2}^{2})$,

$\gamma_{1j}\sim N(0,G_{1})$,

$\gamma_{2j}\sim N(0,G_{2})$.

The vectors $B_{1}$ and $B_{2}$ represent the fixed effects, while $\gamma_{1}$ and $\gamma_{2}$ denote the random effects. The design matrices for the fixed and random effects are $X_{1j}$,$X_{2j}$,$Z_{1j}$ and $Z_{2j}$, respectively.

To examine the association between nutritional status and anemia status, a bivariate linear mixed model can be expressed as:

(3) $$Y_{j}\;=\;BX_{j}\;+\;Z_{j}\gamma_{j}\;+\;\varepsilon_{j}$$

Here, $\varepsilon_{i}\sim N(0,\Sigma_{i})$ and $\gamma_{i}\;\sim N(0,G_{i})$ where $X_{j}\;=\;\left(\begin{array}{cc} X_{1j} & 0\\ 0 & X_{2j} \end{array}\right)$, $Z_{j}\;=\;\left(\begin{array}{cc} Z_{1j} & 0\\ 0 & Z_{2j} \end{array}\right)$, $B\;=\;\left(\begin{array}{c} b_{1}\\ b_{2} \end{array}\right)$ and $\gamma \;=\;\left(\begin{array}{c} \gamma_{1}\\ \gamma_{2} \end{array}\right)$. The covariance of measurement errors and random effects is defined by $\Sigma \;=\;\left(\begin{array}{cc} \sigma_{1}^{2} & 0\\ 0 & \sigma_{2}^{2} \end{array}\right)$ and $G\;=\;\left(\begin{array}{cc} G_{1} & G_{12}\\ G_{21} & G_{2} \end{array}\right)$.

In this study, matrix G was utilized as the covariance matrix of the joint model, where $G_{1}$ and $G_{2}$ represent the variance components of nutritional status and anemia status, respectively. $G_{12}$ and $G_{21}$ are the denote the correlation components between nutritional status and anemia status.

The likelihood function of the joint responses was obtained under the conditional independence assumption using a maximum likelihood approach [2,18]. Due to the lack of closed-form integrals, an approximation based on Gaussian quadrature was employed, found to be consistent and asymptotically normally distributed.

A shared or correlated child-level random effect was included to model dependence between the two outcomes measured in the same child. A separate country-specific random intercept was included to account for clustering of children within countries and unobserved country-level heterogeneity.

The multivariate joint model offers several advantages over separate models, including better control of type I error rates, increased efficiency in parameter estimates, and the ability to address intrinsically multivariate questions [2,16]. Validation of the fitted model involves ensuring correct specification of linear systematic components, appropriate use of the link function, and identification of outliers. According to literature findings, the joint model using the generalized linear model is deemed the most suitable, considering the correlation between the response variables [2,16,19,20].

The equation of the joint generalized linear mixed model for the nutritional status and anemia status of each child under five years of age can be expressed as:

$$\left(\begin{array}{c} Nutrition\\ Anemia \end{array}\right)=\;\left(\begin{array}{c} b_{10}+b_{11}age\;of\;child+b_{12}gender\;of\;child+\ldots b_{124}household\;wealth\\ b_{20}+b_{21}age\;of\;child+b_{22}gender\;of\;child+\ldots b_{224}household\;wealth \end{array}\right)+\left(\begin{array}{c} \varepsilon_{1}\\ \varepsilon_{2} \end{array}\right)$$

## 5. Results

Multivariable analysis was conducted using the PROC GLIMMIX procedure in SAS 9.4 to jointly model the two binary outcomes using a binomial distribution and logit link. Fixed-effect estimates were obtained on the log-odds scale and exponentiated to produce adjusted odds ratios (aORs). The corresponding lower and upper confidence limits were also exponentiated to obtain 95% confidence intervals (CIs) for the aORs.

The data had a hierarchical structure, with study participants nested within Gabon, The Gambia, Liberia, Mauritania, and Nigeria. To account for unobserved country-level heterogeneity and the correlation among observations from the same country, the model included a country-specific random intercept. This specification allowed the baseline level of each outcome to vary across countries because of differences in socioeconomic conditions, demographic composition, epidemiological profiles, health-system contexts, and other unmeasured country-level factors.

Survey timing was not modelled separately because each country contributed a single survey round and the survey periods were closely associated with country. Consequently, the country-specific random intercept may capture some variation related to survey timing, but country and period effects cannot be completely disentangled. This issue has been acknowledged as a limitation of the study.

The model estimated the adjusted associations between selected maternal, household, dietary, and child-level characteristics and the jointly modelled childhood malnutrition and anaemia outcomes. The aORs, their corresponding 95% CIs, and *p*-values are presented in Table 1.

At the 5% significance level, the factors significantly associated with the outcomes were maternal education, drinking-water source, type of toilet facility, household access to electricity, type of cooking fuel, household wealth, type of mosquito net used during the previous night, consumption of fortified food, child age, child sex, and fever during the two weeks preceding the survey. No statistically significant evidence of interaction was identified among the explanatory variables included in the model.

Maternal educational attainment was significantly associated with childhood malnutrition and anaemia. Compared with children whose mothers had no formal education, children whose mothers had completed secondary education had 8.1% lower adjusted odds of the outcomes (aOR = 0.919, 95% CI: 0.855–0.989; *p* = 0.024). The association was stronger among children whose mothers had attained higher education, who had 20.5% lower adjusted odds than children whose mothers had no formal education (aOR = 0.795, 95% CI: 0.696–0.908; *p* = 0.001). Primary education was not significantly associated with the outcomes (aOR = 0.991, 95% CI: 0.923–1.064; *p* = 0.796). These findings indicate that the association between maternal education and the outcomes was most evident at the secondary and higher levels of education.

Household drinking-water source was also associated with the study outcomes. Relative to children living in households with piped water, children from households that relied on well water had 15.1% higher adjusted odds of malnutrition and anaemia (aOR = 1.151, 95% CI: 1.075–1.233; *p* < 0.0001).

Bottle or sachet water (aOR = 0.826, 95% CI: 0.578–1.181; *p* = 0.294), natural water sources (aOR = 1.044, 95% CI: 0.950–1.147; *p* = 0.369), tanker water (aOR = 0.944, 95% CI: 0.807–1.104; *p* = 0.474), and other water sources (aOR = 1.122, 95% CI: 0.956–1.318; *p* = 0.159) were not significantly associated with the outcomes after adjustment for the other variables in the model.

Sanitation conditions showed a similar pattern. Children living in households with pit latrines had 14.3% higher adjusted odds of malnutrition and anaemia than children living in households with flush toilets (aOR = 1.143, 95% CI: 1.055–1.237; *p* = 0.001). Other types of toilet facilities were not significantly associated with the outcomes (aOR = 1.053, 95% CI: 0.962–1.153; *p* = 0.266).

Household access to electricity was significantly associated with the outcomes. Children living in households without electricity had 12.3% lower adjusted odds of malnutrition and anaemia than children living in households with electricity (aOR = 0.877, 95% CI: 0.814–0.945; *p* = 0.001). This counterintuitive estimate represents an adjusted association conditional on the other variables included in the model and should not be interpreted as evidence that the absence of electricity has a protective effect.

The type of cooking fuel used by the household was significantly associated with the outcomes. Compared with households that used wood, the use of electricity for cooking was associated with 24.4% lower adjusted odds of malnutrition and anaemia (aOR = 0.756, 95% CI: 0.575–0.995; *p* = 0.046). Children living in households that used gas similarly had 10.7% lower adjusted odds than those living in households that used wood (aOR = 0.893, 95% CI: 0.825–0.967; *p* = 0.005).

The category in which no food was cooked was not significantly associated with the outcomes (aOR = 0.506, 95% CI: 0.206–1.242; *p* = 0.137). The wide confidence interval indicates comparatively low precision, potentially reflecting a small number of observations in this category.

Household residence, sex of the household head, and age of the household head were not independently associated with malnutrition and anaemia after adjusting for the other covariates.

Rural residence was associated with 6.6% higher adjusted odds than urban residence, although the association did not reach the 5% significance level (aOR = 1.066, 95% CI: 0.996–1.141; *p* = 0.066). Similarly, children living in female-headed households did not differ significantly from those living in male-headed households (aOR = 0.954, 95% CI: 0.891–1.021; *p* = 0.175). None of the household-head age categories differed significantly from the reference group.

A clear socioeconomic gradient was observed across the household wealth categories. Using children from the richest households as the reference group, the adjusted odds of malnutrition and anaemia increased progressively as household wealth decreased.

Children from the poorest households had 59.5% higher adjusted odds than children from the richest households (aOR = 1.595, 95% CI: 1.399–1.819; *p* < 0.0001). Children from poorer households had 40.4% higher adjusted odds (aOR = 1.404, 95% CI: 1.237–1.592; *p* < 0.0001), while children from middle-wealth households had 32.4% higher adjusted odds (aOR = 1.324, 95% CI: 1.183–1.481; *p* < 0.0001). Children in the richer category had 19.0% higher adjusted odds than those in the richest category (aOR = 1.190, 95% CI: 1.073–1.319; *p* < 0.0001).

The magnitude and ordering of these estimates demonstrate a consistent wealth-related gradient, with the greatest adjusted odds observed among children in the poorest households and progressively smaller associations as household wealth approached the richest category.

The type of mosquito net under which the child slept during the previous night was significantly associated with the outcomes. Using children who slept under untreated nets only as the reference group, children who did not sleep under a net had 21.2% higher adjusted odds of the outcomes (aOR = 1.212, 95% CI: 1.048–1.400; *p* = 0.009). Children who slept under treated nets only also had higher adjusted odds than those who slept under untreated nets only (aOR = 1.177, 95% CI: 1.024–1.353; *p* = 0.022).

The estimate for children who reportedly slept under both treated and untreated nets was not statistically significant and had a wide confidence interval (aOR = 0.718, 95% CI: 0.163–3.158; *p* = 0.661), indicating considerable uncertainty. Household ownership of a mosquito net was not independently associated with the outcomes (aOR = 0.994, 95% CI: 0.920–1.075; *p* = 0.888).

Household ownership of a mosquito net was not independently associated with the outcomes (aOR = 0.994, 95% CI: 0.920–1.075; *p* = 0.888). Thus, the findings differed between the measure of household mosquito-net ownership and the reported type of net under which the child slept during the previous night.

Among the child-feeding variables included in the model, consumption of fortified food was significantly associated with malnutrition and anaemia. Children who had consumed fortified food had 12.6% lower adjusted odds than children who had not consumed fortified food (aOR = 0.874, 95% CI: 0.795–0.962; *p* = 0.006).

The consumption of milk, formula, tubers, eggs, and meat was not significantly associated with the outcomes after adjustment for the other covariates. Consumption of eggs was associated with 8.3% lower adjusted odds, but the association did not reach the conventional 5% significance level (aOR = 0.917, 95% CI: 0.838–1.004; *p* = 0.061).

No statistically significant associations were found for milk consumption (aOR = 1.004, 95% CI: 0.937–1.076; *p* = 0.911), formula consumption (aOR = 1.039, 95% CI: 0.947–1.139; *p* = 0.419), tuber consumption (aOR = 1.026, 95% CI: 0.955–1.103; *p* = 0.483), or meat consumption (aOR = 0.942, 95% CI: 0.869–1.021; *p* = 0.147).

Child age was strongly associated with the study outcomes. Compared with children in the youngest age group, children aged 12–23 months had 41.8% higher adjusted odds of malnutrition and anaemia (aOR = 1.418, 95% CI: 1.331–1.510; *p* < 0.0001). This represented the largest age-related association observed in the model.

The adjusted odds remained significantly elevated in all older age groups. Children aged 24–35 months had 26.5% higher adjusted odds than the youngest children (aOR = 1.265, 95% CI: 1.154–1.387; *p* < 0.0001). The corresponding increases were 27.5% among children aged 36–47 months (aOR = 1.275, 95% CI: 1.184–1.372; *p* < 0.0001) and 29.6% among children aged 48–59 months (aOR = 1.296, 95% CI: 1.201–1.398; *p* < 0.0001).

Although children in every age group above 12 months had higher adjusted odds than the youngest group, the association was strongest among children aged 12–23 months. The estimates for the three older age groups were comparatively similar, with aORs ranging from 1.265 to 1.296.

Child sex was independently associated with the outcomes. Female children had 6.1% lower adjusted odds of malnutrition and anaemia than male children (aOR = 0.939, 95% CI: 0.895–0.985; *p* = 0.010).

Children who experienced fever during the two weeks preceding the survey had 11.3% higher adjusted odds of malnutrition and anaemia than children who did not experience fever during this period (aOR = 1.113, 95% CI: 1.044–1.188; *p* = 0.001).

Recent cough was not significantly associated with the outcomes (aOR = 0.972, 95% CI: 0.907–1.042; *p* = 0.428). Similarly, not visiting a health facility during the preceding 12 months was not independently associated with malnutrition and anaemia after adjusting for the other variables in the model (aOR = 1.018, 95% CI: 0.962–1.078; *p* = 0.540).

Overall, the largest increase in the adjusted odds of malnutrition and anemia was observed among children from the poorest households, followed by children aged 12 to 23 months. Elevated adjusted odds were also found among children living in households that used well water, households with pit latrines, children who did not sleep under a mosquito net, and children who had experienced recent fever.

Conversely, higher maternal education, the use of electricity or gas rather than wood for cooking, consumption of fortified food, and being female were associated with lower adjusted odds of the outcome. A consistent inverse wealth gradient was evident, while the age-related results indicated that children aged 12 to 23 months were particularly vulnerable. Table 1 presents the adjusted odds ratios and 95% confidence intervals for the statistically significant associations.

## 6. Discussion

Malnutrition and anemia remain major public health concerns across the selected African countries of Gabon, The Gambia, Liberia, Mauritania, and Nigeria, where they contribute substantially to childhood illness and mortality. Despite interventions by national governments, non-governmental organisations, and humanitarian agencies to strengthen healthcare policies and expand access to essential services, both conditions remain prevalent. Their continued burden highlights the need for more focused, preventive, and context-specific strategies that address the underlying determinants of child health and nutrition [21,22].

The study identified a range of factors significantly associated with childhood malnutrition and anemia. These included maternal age and educational attainment, household drinking-water source, sanitation facilities, electricity access, cooking fuel, and wealth status, as well as child-level characteristics such as age, sex, consumption of fortified foods, and recent fever. Collectively, these findings demonstrate the complex and multifaceted nature of childhood malnutrition and anemia. Reducing the burden of these conditions therefore requires integrated, context-specific interventions that address socioeconomic and environmental disadvantages alongside children’s nutritional and healthcare needs.

Despite the limited data on the determinants of malnutrition and anemia in Gabon, Gambia, Liberia, Mauritania, and Nigeria, particularly in recent years, the findings of this study align with previous research in other regions. For example, ref. [2] utilized a joint model to identify factors associated with malnutrition and anemia in children under five in Angola, Senegal, and Malawi, highlighting child gender, maternal education, and wealth index as significant determinants—factors also identified in this study. Similarly, ref. [23] using a multivariate joint model on data from Lesotho, found child age to be a significant determinant, consistent with the results presented here. Further, ref. [24] examined anemia and stunting in various African regions, identifying family wealth, maternal education, and living environments as key factors, supporting this study’s conclusions. In addition, ref. [25] conducted a study using a Bayesian hierarchical mixed model with a stochastic partial differential equation to identify risk factors of anemia and malnutrition in Ethiopia. The study revealed gender of the child and maternal education to be significant risk factors, which is in alignment with this current study’s findings. Furthermore, ref. [26] conducted a study on children under five in Rwanda, leveraging off a bivariate binary logistic regression model to assess the association between undernutrition and anemia given the effect of other predictors, the study highlighted that the water source and the presence of a fever were significant risk factors, which further supports this study.

However, it is noteworthy that both [23] and [24] found no significant relationship between malnutrition and variables such as access to adequate toilet facilities, electricity, drinking water source, recent fever incidence, and fortified food intake—factors that were identified as significant in the present study.

The findings of this paper highlight the critical role of primary healthcare interventions in the recognition and management of malnutrition and anemia. In regions with a high prevalence of these conditions, regular workshops and community outreach programs should be prioritized. These initiatives could take place in healthcare facilities during wait times or in collaboration with community leaders in high-traffic areas like places of worship, shopping centers, and grant collection points. Such programs are crucial for raising awareness and educating the public about prevention, early detection, and management of malnutrition and anemia [27].

To effectively combat malnutrition and anemia in West African countries, targeted policy interventions are essential. Routine hemoglobin monitoring during antenatal care visits for at-risk mothers—particularly younger or less-educated women—should be implemented. This would enable the early detection of anemia, allowing for timely treatment and reducing pregnancy-related complications. Additionally, baseline iron studies at the first antenatal care visit for at-risk mothers would further aid in identifying iron deficiency early, allowing for personalized treatment plans to prevent anemia from worsening [28,29].

Healthcare facilities must establish standardized protocols for treating anemia, especially in at-risk mothers. These protocols should include clear guidelines for escalating care, such as moving from oral to intravenous iron supplementation based on the severity of the condition. Effective collaboration between healthcare facilities and adequate resource allocation are crucial to ensure proper treatment is available [28,30].

Addressing environmental risk factors, such as poor sanitation and lack of electricity, is another priority. Community health workers can regularly visit at-risk families, identifying early signs of malnutrition and anemia and ensuring they receive the necessary care. Governments should also consider providing social grants to primary caregivers in low-income households to alleviate financial stress and reduce the risk of malnutrition [31,32,33].

When children present with both anemia and fever, healthcare providers should adhere to the WHO’s ten steps for managing malnutrition. Infections should be ruled out before initiating iron supplementation to improve health outcomes and reduce mortality. These strategies would significantly enhance the region’s capacity to manage malnutrition and anemia effectively [34,35].

High-risk pregnancies, particularly among teenagers and women in low-income households, should be identified early and flagged for additional support. Access to confidential, reliable contraception is essential for young women to prevent unplanned pregnancies. High-risk mothers could also benefit from prenatal classes focusing on hygiene, nutrition, child development, deworming, vaccinations, and the signs of malnutrition and anemia. These mothers should be monitored by community caregivers, who can provide ongoing counseling and support, easing the burden of frequent clinic visits. Partnerships between local governments and nonprofit organizations are also necessary to ensure families have access to fortified foods, food parcels, and high-protein supplements [36].

Technology can play a pivotal role in addressing malnutrition and anemia by providing high-risk mothers with access to webinars or mobile health applications, which can offer antenatal care guidelines, reminders for clinic visits, and prompts for prenatal supplements. Such tools can help overcome literacy barriers by providing essential health information in an accessible format. These technological solutions can also offer guidance on safe water use and hygiene practices, particularly for families without access to piped water or sanitation services [37,38].

Addressing malnutrition and anemia in West Africa requires a comprehensive approach. A combination of primary healthcare interventions, community outreach, prenatal education, and the use of technology can significantly reduce the burden of these conditions in the region, ultimately improving public health outcomes.

## 7. Conclusions

Using a joint modelling approach, this study examined factors associated with childhood malnutrition and anemia in Gabon, The Gambia, Liberia, Mauritania, and Nigeria. Maternal, child, household, and environmental characteristics were significantly associated with the two outcomes, including maternal age and education, child age and sex, household wealth, access to water and sanitation, electricity, cooking fuel, fortified-food consumption, and recent fever. The observed association between malnutrition and anemia supports their integrated assessment rather than their consideration as entirely separate child-health conditions. Interventions should therefore combine nutritional and healthcare support with measures addressing the socioeconomic and environmental disadvantages experienced by vulnerable households.

## 8. Strengths and Limitations

The study effectively employs joint modeling, a method well-suited for analyzing the association between anemia and malnutrition, which is essential for understanding the interactions and overlapping risk factors of these conditions. This approach surpasses traditional separate models by providing a comprehensive view of co-morbid factors.

By utilizing a joint modeling framework, this research achieves more accurate parameter estimation for anemia and malnutrition determinants, ultimately offering a more reliable foundation for policy recommendations.

The study spans multiple West African countries, specifically Gabon, Gambia, Liberia, Mauritania, and Nigeria, providing insights into the socio-demographic and health-related drivers of anemia and malnutrition across diverse settings. This broad scope enriches the findings and supports region-specific policy and intervention development.

The study’s reliance on cross-sectional data limits its ability to track trends and causal relationships over time, which is crucial for understanding the persistence of anemia and malnutrition.

Longitudinal studies would be essential to capture long-term trends and provide a more nuanced view of the interplay between anemia, malnutrition, and related socio-environmental factors, enabling more targeted and effective interventions.

## Figures and Tables

**Table 1:** Parameter estimates of factors associated with malnutrition and anemia among children (0–59 months) in Gabon (2019–2021), Gambia (2019–2020), Liberia (2019–2020), Mauritania (2019–2021), and Nigeria (2021).

Variable Name	Estimate	aOR	95% CI		Pr > |t|
Residence_type (Ref = Urban)					
Rural	0.064	1.066	0.996	1.141	0.066
Mother_highest_education (Ref = No Education)					
Primary	−0.009	0.991	0.923	1.064	0.796
Secondary	−0.084	0.919	0.855	0.989	0.024
Higher	−0.229	0.795	0.696	0.908	0.001
Source of drinking water (Ref = Piped)					
Bottle/Sachet	−0.191	0.826	0.578	1.181	0.294
Natural Sources	0.043	1.044	0.950	1.147	0.369
Tanker	−0.057	0.944	0.807	1.104	0.474
Well	0.141	1.151	1.075	1.233	<0.0001
Other	0.115	1.122	0.956	1.318	0.159
Type of toilet facility (Ref = Flush)					
Pit	0.133	1.143	1.055	1.237	0.001
Other	0.051	1.053	0.962	1.153	0.266
Household has electricity (Ref = Yes)					
No	−0.132	0.877	0.814	0.945	0.001
Gender of the head of the household (Ref = Male)					
Female	−0.047	0.954	0.891	1.021	0.175
Age of the head of household (Ref ≤ 18)					
<25	−0.188	0.829	0.494	1.390	0.477
<35	−0.208	0.812	0.488	1.353	0.424
<45	−0.232	0.793	0.475	1.322	0.374
<55	−0.229	0.795	0.476	1.330	0.382
<65	−0.267	0.766	0.458	1.280	0.309
>65	−0.201	0.818	0.488	1.372	0.445
Type of cooking fuel (Ref = Wood)					
Electricity	−0.279	0.756	0.575	0.995	0.046
Gas	−0.113	0.893	0.825	0.967	0.005
No food cooked	−0.681	0.506	0.206	1.242	0.137
Wealth Index (Ref = Richest)					
Poorest	0.467	1.595	1.399	1.819	<0.0001
Poorer	0.339	1.404	1.237	1.592	<0.0001
Middle	0.281	1.324	1.183	1.481	<0.0001
Richer	0.174	1.190	1.073	1.319	<0.0001
Slept under a net last night (Ref = only untreated nets)					
No net	0.192	1.212	1.048	1.400	0.009
Only treated nets	0.163	1.177	1.024	1.353	0.022
Both treated and untreated	−0.332	0.718	0.163	3.158	0.661
Visted a health facility in the last 12 months (Ref = Yes)					
No	0.018	1.018	0.962	1.078	0.540
Gave the child milk (Ref = No)					
Yes	0.004	1.004	0.937	1.076	0.911
Gave the child formula (Ref = No)					
Yes	0.038	1.039	0.947	1.139	0.419
Gave the child fortified food (Ref = No)					
Yes	−0.135	0.874	0.795	0.962	0.006
Gave the child tubers (Ref = No)					
Yes	0.026	1.026	0.955	1.103	0.483
Gave the child eggs (Ref = No)					
Yes	−0.087	0.917	0.838	1.004	0.061
Gave the child meat (Ref = No)					
Yes	−0.060	0.942	0.869	1.021	0.147
Has a mosquito net (Ref = Yes)					
No	−0.006	0.994	0.920	1.075	0.888
Age of the child (Ref = 0 to 12 months)					
12 to 24 months	0.349	1.418	1.331	1.510	<0.0001
24 to 36 months	0.235	1.265	1.154	1.387	<0.0001
36 to 48 months	0.243	1.275	1.184	1.372	<0.0001
48 to 60 months	0.259	1.296	1.201	1.398	<0.0001
Gender of the child (Ref = Male)					
Female	−0.063	0.939	0.895	0.985	0.010
Fever in the last 2 weeks (Ref = No)					
Yes	0.107	1.113	1.044	1.188	0.001
Cough in the last 2 weeks (Ref = No)					
Yes	−0.028	0.972	0.907	1.042	0.428

## Data Availability

The dataset analyzed during this study is not publicly available. Additionally, further information about the data and conditions for access are available upon registration and request to DHS program—http://dhsprogram.com.
